# The use of Karnofsky Performance Status (KPS) as a predictor of 3 month post discharge mortality in cirrhotic patients 

**Published:** 2018

**Authors:** Muhammad Ali Khalid, Inamullah Khan Achakzai, Shoaib Ahmed Khan, Zain Majid, Farina M Hanif, Javed Iqbal, Syed Mudassir Laeeq, Nasir Hassan Luck

**Affiliations:** *Department of Hepatogastroenterology, Sindh Institute of Urology and Transplantation, (SIUT) Karachi, Pakistan *

**Keywords:** Karnofsky Performance Status (KPS), Cirrhosis, 3 months mortality

## Abstract

**Aim:**

Is Karnofsky Performance Status (KPS) a predictor of 3 month post discharge mortality in cirrhotic patients?

**Background:**

Cirrhotic patients often experience an abrupt decline in their health, which often leads to frequent hospitalization and can cause morbidity and mortality. Various models are currently used to predict mortality in cirrhotics however these have their limitations. The Karnofsky Performance Status (KPS) being one of the oldest performance status scales, is a health care provider–administered assessment that has been validated to predict mortality across the elderly and in the chronic disease populations.

**Methods:**

We used the KPS performance status scale to envisage short-term mortality in cirrhotic and HCC patients who survive to be discharged from hospital.

**Results:**

Our study showed that KPS one week post-discharge, child pugh score, hospital stay, international normalized ratio, serum albumin, total bilirubin and serum creatinine showed statistical significance on univariate analysis. On multivariate analysis, KPS was found to be statistical significant predictor of 3-month mortality.

**Conclusion:**

Hence KPS can be utilized to identify cirrhotic patients at risk of 3-month post discharge mortality.

## Introduction

 Hospitalization is a marker of poor outcomes including readmission and death. Patients with cirrhosis experience abrupt deterioration in their health that leads to repeated hospitalizations along with increased morbidity and mortality (1-3). Currently, the models used to predict mortality in cirrhotics are liver-specific and kidney-specific prognostic indicators such as the Model for End-Stage Liver Disease (MELD) score (4). However the MELD score has several limitations (5-7) one of them being its lack of ability to account for an individual’s performance status. 

It is now a well-known fact that performance status and the linked concept of infirmity are strong predictors of adverse outcomes in patient populations (8-14) including cirrhosis (5-18) and it often outperforming established prognostic markers (19-20).

The Karnofsky Performance Status (KPS) is a health care provider–administered assessment that takes 1-2 minutes to assign a patient to one of the 10 categories (ranging from 0 [dead] to 100 [normal activity, no evidence of disease]). It is one of the oldest performance status scales (21) and has been validated to predict mortality across elderly and chronic disease populations (22-24). Orman et al identifying the KPS, independent of the liver function, as a predictor of liver transplant waiting list mortality (16). Thus KPS has been shown to be an important, user-friendly screening modality for an additional general risk stratification that can be readily administered in any clinical setting. In addition to being practical and easy to use, the validity and reliability of the KPS are well established (24-26).

Therefore the need to establish a practical prognostic model that could identify those at the highest risk of 3-months mortality lead us to validate the KPS as a prognostic predictor in our patient populations (those with cirrhosis and hepatocellular carcinoma).Since this would assist us in selecting patients who need a more intensive follow-up and consideration of early liver transplant when available.

Furthermore, an assessment of the probability of survival could help us update the type of palliative support that is provided to patients.

**Table 1:** Analysis of the various parameters seen in our patients

Variable				p-value
Age, years, mean±SD (range)	42.83 ± 19.7 ( 20 – 75)			0.38
Gender, n (%)	Male :	70 (64.8 %)		0.31
	Female:	38 (35.2 %)		
Child Class, n (%)	CTP A:	33 (30.6 %)		<0.0001
	CTP B :	46 (42.6%)		
	CTP C :	29 ( 26.9 % )		
HCC presence, n (%)	50 (46.3 %)			0.23
1 week Hospital stay, n (%)	88 (81.5 %)			0.005
KPS one week after discharge, n (%)	Low		23	0.00
	intermediate		49	
	High		36	
international normalized ratio, mean±SD	1.52±0.64 ( 1 – 6.2)			<0.0001
Albumin, mean±SD	2.8 ±0.73 (1 – 4.7)			<0.0001
Total bilirubin, mean±SD	3.9 ±6.9 (0.22 – 43.3 )			<0.0001
alanine aminotransferase, mean±SD	95.3± 141.4 ( 4 – 800)			0.12
Alkaline Phosphatase, mean±SD	171.5 ± 110.1 ( 2 – 731 )			0.32
Gamma- Glutamyl transferase, mean±SD	118.3 ± 140.3 ( 1 – 981)			0.43
Serum Sodium, mean±SD	133.8 ± 14.3 ( 2 – 145 )			0.93
Serum Creatinine, mean±SD	1.29 ± 1.2 (0.26 – 9.5 )			0.009

## Methods

Cirrhotic patients of either gender were prospectively enrolled non-electively in this study conducted at the Department of Hepatogastroenterology, Sindh Institute of Urology and Transplantation (SIUT), Karachi, Pakistan, a large tertiary care centre located in Pakistan’s largest city Karachi. These patients were followed for 3 months throughout their hospitalization or after discharge by using systematic phone calls, during outpatient visits and interviewed to evaluate for outcomes either within the hospital or elsewhere (1,27)

Patients were recruited from June 2016 until July 2017. A diagnosis of cirrhosis was established by endoscopic or radiological evidence of portal hypertension or cirrhosis, compatible biopsy findings, and/or signs of hepatic decompensation, including hepatic encephalopathy (HE), jaundice, variceal bleeding, and ascites. Patients who failed to give informed consent, those who were transplanted during hospitalization and those with metastatic cancer (excluding Hepatocellular Carcinoma), human immunodeficiency virus, or inability to obtain KPS assessment at 1 week after hospital discharge were excluded.

Data were collected regarding patient demographics, liver disease etiology and severity (MELD and Child-Pugh scores), admission variables (admission indication, cirrhosis complications and organ failures occurring during hospitalization, length of stay), and discharge variables (laboratory results, medications). At 1 week post discharge, the research assistant assessed the patient’s KPS using questions directed to the patient and the caregiver in a telephonic interview. The KPS score was categorized into low (score 10-40), intermediate (50-70), and high (80- 100) (16). Subjects were followed for 3 months post discharge systematically to evaluate health outcomes. The collected data were entered into SPSS (version 20.0) and analyzed by the researcher. Mean and standard deviation were calculated for continous variables. Frequency and percentages were calculated for categorical variables. Models for the prediction of 3- month mortality were based on variables measured at baseline, during the index stay, on the date of discharge from the index admission, and KPS at 1 week post discharge. A p value < 0.05 was considered significant. 

## Results

The total number of patients enrolled were 108 out of which 64.8% were predominantly males. The mean age noted was 42.83 years. Most patients fell in CTP class B (42.6%).Hepatitis B virus (HBV) related and Hepatitis D related CLD was seen in 17 (15.8 %) and in 1 (0.92 %) patients respectively. Twenty six (24.07 %) patients had CLD due to unknown cause. (Seen in Table 1).

Hepatocellular carcinoma (HCC) was present in 50 (46.3 %) patients. Among these patients, 9 (18%) patients had a prior history of HBV infection while the remaining had HCV related CLD. Trans arterial chemoembolization (TACE) was performed in 41 ( 82 %) patients with tumor recurrence being documented in 8 ( 19.5 %) of these patients. 

A 1 week hospital stay was noted in majority of patients i.e., 88 patients (81.5 %) while the rest had a stay more than 2 weeks. According to KPS scoring, 49 (45.4 %) patients fall into intermediate performance group, followed by 36 (33.33 %) patients in high and 23 (21.3 %) patients in low performance group.

The three month post discharge mortality was documented in 44 (40.7 %) patients. The mortality rates for the low, intermediate, and high performance status groups were 95.65 % (22/ 23), 38.7 % (19/ 49), and 8.3 % (3/ 36), respectively.

KPS one week post- discharge (p= 0.00), Child class score (p=0.00), hospital stay (p=0.005), international normalized ratio (p=0.00), serum albumin (p=0.000), total bilirubin (p=0.000), serum creatinine (p=0.009) showed statistical significance on univariate analysis. On multivariate analysis, KPS (p=0.016) was found to be statistical significant predictor of 3-month mortality.

After stratifying data for the presence of HCC, univariate analysis had statistical significance with KPS one week post- discharge (p= 0.00), Child class score (p=0.04), international normalized ratio (p=0.04) and serum albumin (p=0.000). On multivariate analysis, KPS (p= 0.34) and serum albumin (p=0.016) were found to be a statistical significant predictor of 3-month mortality after discharge.

## Discussion

In cirrhotic patients accurate prognostication is indispensable because it guides us to prescribe the type and decide about the frequency of clinical care and helps us inform patients and their families about their possible outcomes. To our information, this is the first single center prospective study done in Pakistan using a performance status scale(KPS) to envisage short-term mortality in cirrhotic and HCC patients who survive to be discharged from hospital.

 Our study is in accord with Tandon et al (28) who also documented similar finding in their study by using the same KAM model. This also extends the results of the recently done retrospective study done by Orman et al.^16^ and Tapper et al, (15) identified activities of daily living score done at hospital admission as a predictor of either 90-day in-hospital or post discharge mortality.

In routine clinical practice the addition of the KPS has been found to be highly reproducible and predictive in cirrhosis seen during outpatient visits, even when compared to the other more lengthy performance status evaluations such as the Fried Frailty Scale and the Short Physical Performance Battery (18).

As estimated of a hospitalized population, the scores in our cohort were significantly worse than those noted in the transplant waiting list study by Orman et al. (16) At 1 week post–hospital discharge, 33% of patients were in the high performance status range at 1 week post–hospital discharge. Forty- five percent had intermediate scores, reflecting incapability to work and a requirement of support for personal needs. Only 21% of patients had a low KPS score, consistent with lack of ability to care for oneself and the need for the equivalent of institutional care. The proper provision of support in this area may be a key factor in reducing the astonishing rates of rehospitalization in this population (1). The poor performance status of this group supports the significance of a multidisciplinary approach prior to discharge these patients. We have also seen similar findings in HCC group i.e. those who had low KPS scores at 1 week, they have a high 3 months mortality in our follow up.

A low 1 week post discharge KPS score was predicted by child class score, hospital stay, international normalized ratio (INR), serum albumin, total bilirubin, serum creatinine. A more meticulous assessment of potentially adjustable factors that impact performance status in hospitalized patients will be an imperative area of focus for follow-on studies. As has been eminent in studies of functional decline occurring during hospitalization, it is expected that potentially amendable factors such as in-hospital nutritional intake and in hospital mobilization may also play a vital role in predicting discharge performance status (29).

The independent prognostic assessment of performance status highlights the need to incorporate palliative care principles (advanced care planning, goals of care discussions, palliative symptom management) in the management of the bulk of our patients being discharged from hospital. Patients at high peril of death should be assessed for a care plan using integrated palliative management strategies and, if possible, considered for live donor liver transplantation.

In our study post discharge three months mortality was documented in 44 (40.7 %) patients. The mortality rates for the low, intermediate, and high performance status groups were 95.65 %, 38.7 % and 8.3 %, respectively. Furthermore, high risk patients may benefit from earlier follow-up and escalation of their active disease management post discharge (30).

The limitations of our study were that we did not assess the KPS on the day of hospital admission or on the day of discharge and as a result we were not capable to depict a change in the performance status. Secondly it was beyond the extent of our study to evaluate several factors previously related with post hospitalization functional decline, including the prehospitalization functional reserve or the nutrition and mobilization therapy provided in hospital. This can be the focus of follow-on studies. Lastly it was a single centre study and further studies will be needed to validate this association.

Almost 41% of cirrhotic patients who survive until discharge die within 3 months after hospitalization. These patients can be identified using the KPS based performance status score. This easy-to-use measurement is strongly and independently linked with an increased hazard of mortality and could be adopted in practice to lead post discharge early interventions, as well as the integrated provision of vigorous and palliative management strategies.

## Conflict of interests

The authors declare that they have no conflict of interest.
